# Mitochondria and Calcium Homeostasis: Cisd2 as a Big Player in Cardiac Ageing

**DOI:** 10.3390/ijms21239238

**Published:** 2020-12-03

**Authors:** Chi-Hsiao Yeh, Yi-Ju Chou, Cheng-Heng Kao, Ting-Fen Tsai

**Affiliations:** 1 Department of Thoracic and Cardiovascular Surgery, Chang Gung Memorial Hospital, Linko 333, Taiwan; yehccl@cgmh.org.tw; 2 College of Medicine, Chang Gung University, Taoyuan 333, Taiwan; 3 Community Medicine Research Center, Chang Gung Memorial Hospital, Keelung Branch, Keelung 204, Taiwan; 4 Institute of Molecular and Genomic Medicine, National Health Research Institutes, Zhunan 350, Taiwan; yjchou0810@nhri.edu.tw; 5 Center of General Education, Chang Gung University, Taoyuan 333, Taiwan; 6 Department of Life Sciences and Institute of Genome Sciences, National Yang-Ming University, Taipei 112, Taiwan; 7 Institute of Biotechnology and Pharmaceutical Research, National Health Research Institutes, Zhunan 350, Taiwan; 8 Aging and Health Research Center, National Yang-Ming University, Taipei 112, Taiwan

**Keywords:** cardiac ageing, Cisd2, mitochondria-associated membranes (MAMs), mitochondria, calcium homeostasis, energy flexibility

## Abstract

The ageing of human populations has become a problem throughout the world. In this context, increasing the healthy lifespan of individuals has become an important target for medical research and governments. Cardiac disease remains the leading cause of morbidity and mortality in ageing populations and results in significant increases in healthcare costs. Although clinical and basic research have revealed many novel insights into the pathways that drive heart failure, the molecular mechanisms underlying cardiac ageing and age-related cardiac dysfunction are still not fully understood. In this review we summarize the most updated publications and discuss the central components that drive cardiac ageing. The following characters of mitochondria-related dysfunction have been identified during cardiac ageing: (a) disruption of the integrity of mitochondria-associated membrane (MAM) contact sites; (b) dysregulation of energy metabolism and dynamic flexibility; (c) dyshomeostasis of Ca^2+^ control; (d) disturbance to mitochondria–lysosomal crosstalk. Furthermore, Cisd2, a pro-longevity gene, is known to be mainly located in the endoplasmic reticulum (ER), mitochondria, and MAM. The expression level of Cisd2 decreases during cardiac ageing. Remarkably, a high level of Cisd2 delays cardiac ageing and ameliorates age-related cardiac dysfunction; this occurs by maintaining correct regulation of energy metabolism and allowing dynamic control of metabolic flexibility. Together, our previous studies and new evidence provided here highlight Cisd2 as a novel target for developing therapies to promote healthy ageing

## 1. Introduction

Despite the fact that the clinical management of cardiac diseases has improved over the past decades, cardiac disease remains the leading cause of mortality in an ageing population [1]. In particular, as the number of older individuals in populations has increased in many countries, a steep increase in age-related heart failure has begun to represent one of the greatest challenges confronting global health care [2]. In the United States of America, the estimated number of adults that are 20 years of age or older and have suffered from heart failure increased from 5.7 million between 2009 and 2012 to 6.2 million between 2013 and 2016 [1]; this suggests that there is similar higher prevalence all over the world [3]. The limited endogenous capacity of a heart to undergo repair/regeneration results in an accumulating burden of prior insults, which when combined with the structural and functional changes that occur during cardiac ageing, results in diminished cardiac reserves and an elevated risk of heart failure in older populations [4]. The grave prognosis for aged patients who have suffered heart failure is the result of a number of unknown molecular mechanisms that underlie the pathophysiology of cardiac ageing. However, accumulating evidence has shown that mitochondria play a central role in cardiac ageing and age-related cardiac diseases. Mitochondrial dysfunction brings about dysregulated Ca^2+^ homeostasis [5,6], impaired mitophagy [7,8], and metabolic inflexibility [9,10,11]. Healthy mitochondria are essential for cardiomyocytes to maintain a normal functionality in the heart.

Mitochondrial dysfunction is one of the hallmarks of ageing [12]. To meet the high-energy requirement of the heart, mitochondria occupy about 40% of the volume of cardiomyocytes [13]. Ageing affects the functioning of both mitochondria and the endoplasmic reticulum (ER), as well as having an impact on their contact sites in the cardiomyocytes, namely the mitochondria-associated membranes (MAMs). In the past decade, the markedly better understanding of the structures, tethers, and functions of MAMs has revealed the important roles that these structures play in cell physiology and ageing [14]. Four major functions of the membrane contact sites at MAMs have been identified, namely signaling, the regulation of organelle membrane dynamics, lipid transport, and metabolic channeling [14,15,16]. In an aged heart, age-related mitochondrial alterations include reduced mitochondrial Ca^2+^ uptake and diminished buffering capacity against reactive oxygen species (ROS); these result in impaired metabolism, which in turn bring about an increased sensitivity of the heart to stress, as well as compromising cardiac functioning. Therefore, a deeper understanding of mitochondrial and MAM functioning during cardiac ageing is pivotal to ultimately develop novel therapeutic strategies to reduce the burden of ageing.

CISD2 is an evolutionally conserved protein that is mainly located in the mitochondria, ER, and MAMs. The expression level of Cisd2 decreases during natural ageing in many tissues and organs, including the heart, skeletal muscles, liver, brain, and skin. Previously our studies have revealed that Cisd2 knockout results in a premature aging phenotype with a shorten lifespan. Furthermore, Cisd2 deficiency leads to mitochondrial dysfunction, a disruption of cytosolic Ca^2+^ homeostasis, elevated ROS production, and dysregulated autophagy [17,18,19,20,21,22,23]. Conversely, a persistently high level of Cisd2, which can be achieved by transgenic overexpression, is able to reverse age-related cardiac dysfunction [19], Alzheimer’s-related neuronal loss [23], nonalcoholic fatty liver disease [18], and sarcopenia [22]. It does the above by maintaining Ca^2+^ homeostasis, reducing ROS production, and improving metabolic flexibility. Recent genetic evidence highlights Cisd2 as a promising new target for developing novel therapeutics that are aimed at attenuating cardiac ageing and providing a new avenue to geroprotection.

## 2. Defective Mitochondria–Lysosomal Crosstalk during Cardiac Ageing

Cardiovascular disease is the leading cause of mortality among older adults [24]. At the structural level, age-related mitochondrial damage, which includes enlarged organelles, matrix derangement, and loss of cristae of mitochondrial inner membrane, is often found in aged hearts. At the functional level, age-related functional impairment, which includes decreased ATP production, increased ROS generation, and defective quality control, is also evident in aged hearts. All these defects contribute to pathogenesis during age-related heart failure [25]. In addition, during cardiac ageing, defective renewal mechanisms for various cellular constituents preclude the clearance of damaged biomolecules and senescent organelles. For example, accumulation of lipofuscin and degenerated mitochondria is evident in aged cardiac muscles. It should be noted that lipofuscin is considered to be one of the age-associated pigments and is found in skin, neurons, and cardiomyocytes [26,27]. These pigment granules are mainly composed of lipid material and highly oxidized proteins that cannot be digested by the ubiquitin-proteasome system and thus accumulate, primarily in lysosomes. The imbalanced lipid metabolism associated with mitochondria, the compromised enzymatic activity present in the lysosomes, and the presence of impaired autophagy pathways, together with inefficient cellular proteostasis, all seem to contribute to the formation of lipofuscin. Accordingly, the accumulation of nondegraded molecules in the lysosomes, including the deposition of lipofuscin, can be used to indicate the presence of age-related damage to both the mitochondria and lysosomes. These findings suggest that defective mitochondria–lysosomal crosstalk is present during cardiac ageing.

The most clinical relevant disorder related to lipofuscin accumulation is neuronal ceroid lipofuscinosis, which involves the accumulation of autofluorescent materials in cardiomyocytes [28,29]. Patients with neuronal ceroid lipofuscinosis display various clinical manifestations, including ventricular hypertrophy, sinus node dysfunction, and arrhythmia [29]; interestingly, these phenotypes are very similar to those associated with the age-related cardiac phenotype. Furthermore, a previous human study using cardiac samples from autopsy patients has revealed that the accumulation of myocardial lipofuscin directly reflects the chronological age of the heart rather than human cardiac pathology [27].

**Figure 1:**
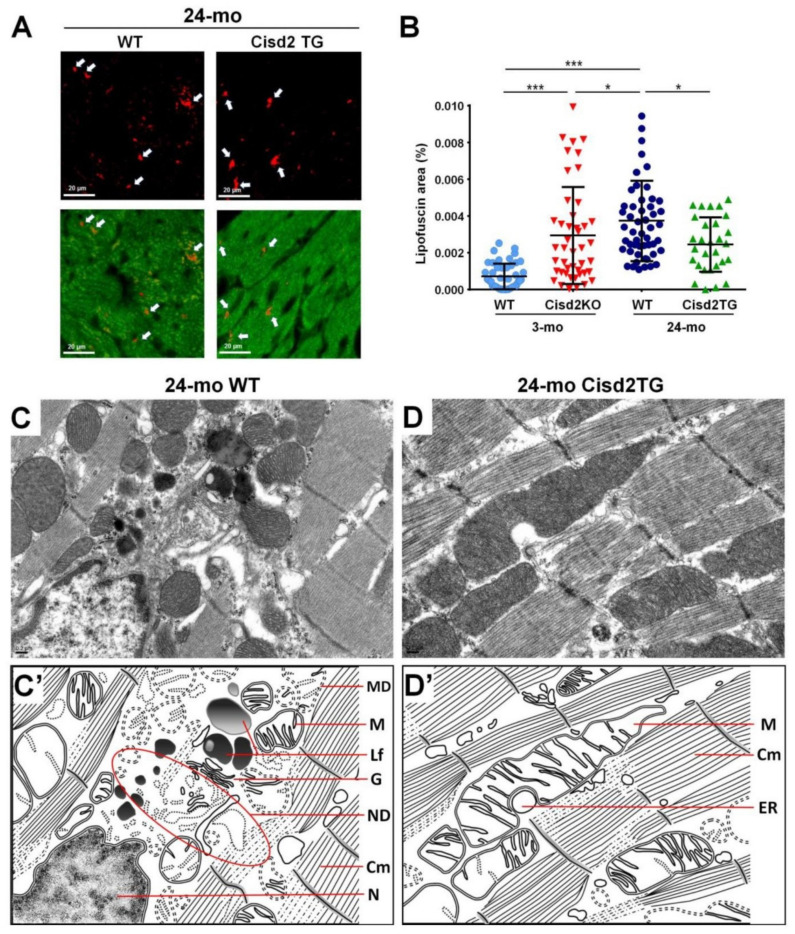
Cisd2 delays cardiac ageing as revealed by reduced lipofuscin accumulation and preserved myocardial ultrastructure. (**A**) Lipofuscin deposits can be clearly identified in cardiac tissues by autofluorescence detected with 330–380 nm excitation light and 420 nm barrier filter of a confocal fluorescence microscope. The red color indicates lipofuscin and green color indicates myocardia. (**B**) There is a significant increase of lipofuscin accumulation during cardiac ageing of WT mice. Intriguingly, a high level of Cisd2 reduces lipofuscin accumulation in Cisd2TG mice, while Cisd2 deficiency significantly elevates lipofuscin accumulation in Cisd2KO mice. The areas of lipofuscin accumulation and the myocardia are measured using Fiji/ImageJ 1.52e (National Institutes of Health, Bethesda, MD, USA). Ten fields per subject are observed at 20× objective magnification, and the lipofuscin accumulation (lipofuscin deposit area/myocardial area) was calculated. * *p* < 0.05, ** *p* < 0.01, *** *p* < 0.001. (**C**,**D**) Transmission electron microscopy (TEM) analysis for the left ventricle of heart. In 24-mo WT mice (**C**), age-associated mitochondria degeneration, lipofuscin accumulation, and necrotic debris of degenerative myofibril and organelles are detectable and easily identified. (**D**) In 24-mo Cisd2TG mice, relatively normal ultrastructures, namely intact mitochondria, ER, and cardiac myofibrils, are found. (**C′**) (**D′**) Schematic presentation for ultrastructure of left ventricle shown in (**C**) and (**D**), respectively. MD: mitochondrial degeneration, Lf: lipofuscin, ND: necrotic debris of degenerative myofibril and organelles, G: Golgi apparatus, M: mitochondria, Cm: cardiac myofibril, ER: endoplasmic reticulum, N: nucleus.

Intriguingly, our studies have shown that Cisd2 can delay cardiac ageing and this is associated with reduced lipofuscin accumulation and preserved myocardial ultrastructure. During the cardiac aging of WT mice, there is a significant increase in lipofuscin accumulation in myocardial tissues. Intriguingly, a high level of Cisd2 reduces lipofuscin accumulation in Cisd2TG mice, which form our long-lived mouse model. Conversely, Cisd2 deficiency significantly elevates lipofuscin accumulation in Cisd2KO mice, which form an accelerated aging mouse model (Figure 1A,B). Moreover, transmission electron microscopy (TEM) analysis has revealed that a high level of Cisd2 expression is able to diminish the accumulation of lipofuscin in cardiomyocytes, to preserve the structural integrity of myofibrils while at the same time ameliorating age-related damage to the ER and mitochondria (Figure 1C,D).

## 3. Cisd2 Deficiency Causes Dysregulation of Intracellular Ca^2+^ Homeostasis

### 3.1. Mitochondria and Cellular Ca^2+^ Homeostasis

Calcium signaling plays a crucial role in many molecular processes and a range of cellular functions. Accumulating evidence indicates that Ca^2+^ strictly controls cellular senescence [6]. An elevation of intracellular Ca^2+^ levels has been observed in response to different types of stresses. Mitochondria, equipped with Ca^2+^ uniporters and a negative membrane potential (about −180 mV), are able to function as a potent Ca^2+^ buffering organelle when there is an elevation in intracellular Ca^2+^ concentration ([Ca^2+^]_i_) [30]. Furthermore, mitochondrial Ca^2+^ signaling increases when an advanced age is reached [31]. The ER and mitochondria are the two major organelles that govern the intracellular storage, sequestration, and release of [Ca^2+^]_i_ [32,33]. An elevation in [Ca^2+^]_i_ is usually attributable to a Ca^2+^ influx from the extracellular milieu via plasma membrane Ca^2+^ channels or from intracellular stores of Ca^2+^ present in the ER [6]. Any release of Ca^2+^ from ER elevates the cytosolic Ca^2+^ and this occurs in combination with ever larger cycles of mitochondrial Ca^2+^ uptake [34]. These increases in mitochondrial Ca^2+^ concentration leads to a drop in mitochondrial membrane potential, which in turn enhances the production of ROS. As a result, oxidative stress increases further, triggering senescence [6], as well as organ dysfunction, during the aging process [31]. Mitochondrial Ca^2+^ uniporter (MCU) and H^+^/Ca^2+^ exchanger LETM1 are the two main transporters responsible for mitochondrial Ca^2+^ uptake [35,36]; on the other hand, NCX (Na^+^/Ca^2+^ exchanger) and H^+^/Ca^2+^ exchanger are the two transporters that export Ca^2+^ back to cytoplasm [30,37].

### 3.2. ER-Mitochondrial Ca^2+^ Dysregulation and Cardiac Ageing

The ER, which consists of a spatially extended membranous network, is often positioned in close proximity to other cellular organelles and these areas form membrane contact sites [38]. The sites of physical interaction and communication between the ER and mitochondria, namely the mitochondria-associated ER membranes (MAMs), represents a platform that is fundamentally important to the modulation of Ca^2+^ homeostasis, autophagy, apoptosis, lipid metabolism, metabolic diseases, and tumor growth; these events are mediated via the exchange of lipids and Ca^2+^ ions [5,16,39,40,41,42]. The conserved structures of MAMs found across eukaryotic phyla are key determinants of cell survival and their functionality, which involves the bidirectional trafficking of factors between the two organelles [43,44]. This means that MAMs are dynamic structures that are sensitive to the physiological conditions within cells [16].

Four major functions have been identified for MAMs. These are (a) control of Ca^2+^ signaling, (b) regulation of mitochondrial division, (c) accommodation of lipid biosynthesis, and (d) coordination of the dynamic interactions between mitochondria and the ER [44]. Importantly, MAMs provide an efficient way for Ca^2+^ traffic to take place between the ER and mitochondria and this allows the creation of a higher Ca^2+^ concentration close to the MAMs compared to the surrounding cytoplasm. The Ca^2+^ released from the ER via the MAMs to the mitochondria means that this high local concentration of Ca^2+^ is able to activate various Ca^2+^-dependent processes. These include the tricarboxylic acid (TCA) cycle, oxidative phosphorylation, the regulation of ROS signaling, cell death, and the stimulation of mitochondrial division [14,44,45,46]. A functional protein complex is essential for the tethering of ER-mitochondria and for maintaining the MAM structure; this complex is formed via inositol 1,4,5-trisphosphate receptors (IP3Rs) at ER, voltage dependent anion-selective channel (VDAC) protein in the mitochondrion, and the chaperone 75 kDa glucose regulated protein (GRP75) [14]. In cardiac and skeletal muscle, the Ca^2+^ channel in the ER, namely the ryanodine receptor (RYR), also allows Ca^2+^ traffic from ER to mitochondria close to the MAMs [47]. Many other tether complexes of MAMs had also been reported [38]. Interestingly, when the hearts of Mfn2KO mice are examined, the contact lengths of MAMs are reduced by 30%; this is accompanied by an increased level of ROS, and reduced cardiac contractility [48]. Using the norepinephrine-induced cardiac hypertrophic model, it has been shown that the distance between the ER and mitochondria is increased and that this is accompanied by impaired Ca^2+^ trafficking between the two organelles [49]. Indeed, alterations in the contact length and gap distance of MAMs had been reported to dysregulate Ca^2+^ signaling and affect cardiac metabolism, thereby accelerating cardiac ageing and promoting the development of cardiac hypertrophy, as well as heart failure [50].

### 3.3. Cisd2 Maintains Ca^2+^ Homeostasis

Cisd2 is mainly located in the mitochondrial outer membrane and the ER. In particular, Cisd2 is highly enriched in the MAMs. Previous studies by our group and other researchers have revealed that the intracellular localization and binding partners of Cisd2 vary with the cell type and/or with the experimental conditions [51]. This scenario helps to explain why different proteins have been found to interact with Cisd2 under a variety of circumstances [18,19,21,22,23,52,53]. For example, in the skeletal muscle, Cisd2 appears to associate with Bcl-2/Bcl-XL and IP3R when participating in the regulation of the ER Ca^2+^ store. Loss of Cisd2 in myocytes results in a dysregulation of Ca^2+^ homeostasis that is accompanied by augmented autophagy and the presence of degenerated ER [52,53]. On the other hand, in the liver, Cisd2 interacts directly with Serca2b, which is a Ca^2+^-pump that transports Ca^2+^ from the cytosol into the ER. Furthermore, investigations have revealed that Cisd2 directly binds to Serca2a in cardiomyocytes. In both cases, this allows there to be control of Ca^2+^ pump activity by these two proteins via modulation of their oxidative modification. This in turn regulates ER Ca^2+^ uptake by Serca2a and Serca2b, which then sustains Ca^2+^ homeostasis in the heart and liver, respectively [18,19]. Finally, in adipocytes, our research has revealed that Cisd2 interacts directly with Gimap5 (GTPase of immune-associated protein 5) on the mitochondrial and ER membranes in order to modulate the Ca^2+^ buffering capacity of mitochondria, thereby maintaining the intracellular Ca^2+^ homeostasis of adipocytes. Loss of Cisd2 increases the cytosolic level of Ca^2+^, and induces Ca^2+^-calcineurin-dependent signaling that inhibits adipogenesis [21].

Mechanistically, one important question is how Cisd2 modulates intracellular Ca^2+^ homeostasis via its interaction with its partner proteins. Here we use Serca2 as an example to illustrate the potential molecular mechanism behind the regulation of Ca^2+^ via Serca2a in the cardiomyocytes and Serca2b in the hepatocytes [18,19]. Three possibilities may explain how the redox status of Serca2a and Serca2b is affected by Cisd2. First, the only defined functional domain of the Cisd2 protein is the protein’s CDGSH domain, which binds a redox-active 2Fe-2S cluster and is oriented toward the cytosol. It seems likely that Cisd2 directly interacts with Serca2 to help maintain Serca2b in a reduced state via the redox capacity of the CDGSH domain present on Cisd2. Second, Cisd2 may cover a part of Serca2 via protein–protein interaction, thus reducing the accessibility of certain tyrosines and cysteines present on Serca2 to attack by enzymes; as a consequence, this may prevent Serca2 from undergoing irreversible oxidative modification (cysteine sulfonation and tyrosine nitration) during oxidative stress. Finally, the redox-active CDGSH domain of Cisd2 may contribute to the maintenance of the general redox status of the cell. This will act to reduce the overall oxidative stress in the cell and indirectly protect Serca2 activity from ROS-mediated and/or RNS-mediated protein oxidation that can bring about a reduction in the protein’s Ca^2+^ pumping activity. These possibilities are not exclusive, but rather all may contribute to maintaining the redox homeostasis of the Serca2b protein [18].

### 3.4. Cisd2 Protects the Interfibrillar and Subsarcolemmal Mitochondria from Age-Related Damage

Mitochondria can be divided into three distinct populations based on their location and proximity to Ca^2+^ release sites [54]. These are, firstly, perinuclear mitochondria (PNMs), which are arranged in clusters and located adjacent to the nucleus; their function is presumably associated with transcription. Secondly, there are subsarcolemmal mitochondria (SSMs), which are also arranged in clusters, but are located just beneath the sarcolemma; their function is presumably associated with the functioning of ion channels and various signaling pathways. Thirdly, there are the interfibrillar mitochondria (IFMs), which are arranged in rows alongside the myofibrils and are located very close to the Ca^2+^ release sites of the SR; their function is presumably to generate ATP that then contributes to the contractile function of myofibrils, as well as to participate in the Ca^2+^ signaling within cardiomyocytes [55].

The IFMs are probably the most susceptible mitochondria to age-related damage. Previous studies have revealed that, during the ageing of cardiomyocytes, there is an increase in mitochondrial ROS and a decrease in antioxidant capacity that leads to an increased accumulation of oxidative damage with the DNA [56]; notably this is almost exclusively associated with the IFMs [57]. The abundance of IFMs and their physically close association with myofilaments suggests that IFMs provide the energy needed for cardiomyocyte contraction and thus have a higher respiratory capacity than SSMs [58]. Therefore, any loss of IFMs is likely to adversely affect muscle contractility by limiting the ATP production required by myosin ATPases. Moreover, the increased ROS level associated with aged IFMs is likely to exacerbate mitochondria-derived oxidant injury to the myofilaments; this in turn will lead to increased fibrosis and/or fiber disarrangement, thereby decreasing the contractile capacity of the heart [59]. Additionally, a previous study has shown that decreased mitophagy is accompanied by mitochondrial fragmentation in aging hearts [60]. Intriguingly, these ultrastructural alterations to the IFMs, namely the presence of fragmented mitochondria, the appearance of disarranged and degenerative myofibrils, and the presence of myofibril/organelle necrotic debris, seem to be absent in 24-mo old Cisd2TG mice, which is not the case in naturally aged 24-mo old WT mice (Figure 1C,D).

**Figure 2:**
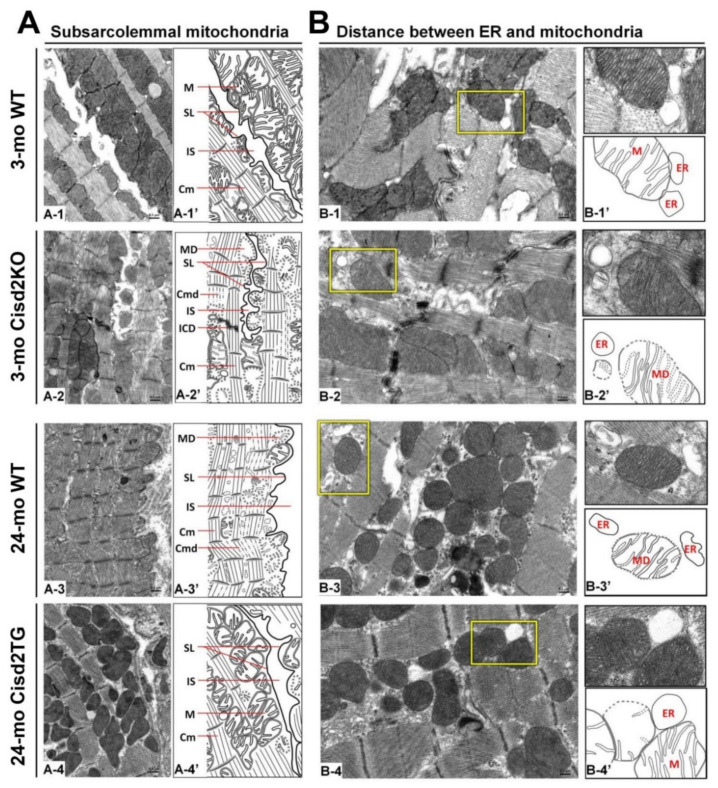
Cisd2 preserves the integrity of subsarcolemmal mitochondria and maintains the ultrastructure of mitochondria-associated ER membrane (MAM). (**A**) Cisd2 preserves the integrity of subsarcolemmal mitochondria and cardiac myofibrils of left ventricle. In 3-mo WT mice, there is an abundance of normal subsarcolemmal mitochondria and normal cardiac myofibrils (A-1 and A-1′). In 3-mo Cisd2KO mice (prematurely aged), notably, a low density of subsarcolemmal mitochondria, and degeneration of mitochondria and myofibrils are found (A-2 and A-2′). In 24-mo WT mice (naturally aged), similar ultrastructural damages are found as those observed in the 3-mo Cisd2KO mice (A-3 and A-3′). Remarkably, in 24-mo Cisd2TG mice, a high level of Cisd2 preserves the density and integrity of subsarcolemmal mitochondria as well as maintains the ultrastructure of cardiac myofibrils (A-4 and A-4′). (A-1′) to (A-4′) are schematic presentations for ultrastructure of left ventricle shown in (A-1) to (A-4). SL: sarcolemma, IS: intercellular space, M: mitochondria, Cm: cardiac myofibril, ICD: intercalated disc, MD: mitochondrial degeneration, Cmd: cardiac myofibril degeneration. (**B**) Cisd2 is essential to maintaining the integrity of MAM. In 3-mo WT mice, at MAM, mitochondria closely attach to ER (B-1 and B-1′). In 3-mo Cisd2KO mice, notably, a longer distance between mitochondria and ER is found; in addition, mitochondria degeneration is also found (B-2 and B-2′). In 24-mo WT mice, a longer distance between mitochondria and ER is found; this is similar to that observed in the 3-mo Cisd2KO mice (B-3 and B-3′). Intriguingly, in 24-mo Cisd2TG mice, a high level of Cisd2 maintains a relatively normal MAM, where the mitochondria closely attach to the ER (B-4 and B-4′). (B-1′) to (B-4′) are schematic presentations for the ultrastructure of left ventricle shown in (B-1) to (B-4). The inset shows a higher magnification to provide a better illustration for MAM. The inset shows a higher magnification of the selected area (yellow squares) of the middle panel to provide a better illustration for MAM.

The function of SSMs is presumably related to ATP production that allows electrolyte and protein transport across the sarcolemma [58]. The exclusive localization of Connexin 43, which is the major component of gap junctions for cell–cell communication, within the SSMs further pinpoints their important role in electrical conductance within the heart [61]. In the naturally aged (24-mo old WT) and prematurely aged (3-mo old Cisd2KO) mice, the SSMs have largely disappeared with only a few degenerating SSMs remaining detectable. This seems to explain the presence of the age-related cardiac conductance abnormalities that occur during ageing (Figure 2A). Remarkably, in the long-lived mice (24-mo old Cisd2TG), a persistently higher level of Cisd2 is able to eliminate age-related damage to the SSMs in the cardiomyocytes; the amount and structural integrity of SSM in the 24-mo old Cisd2TG is similar to that of 3-mo old young WT mice (Figure 2A). This result is consistent with our previous study [19] wherein a higher level of Cisd2 was found to preserve functional mitochondria. This will, in turn, provide a stable MAM microenvironment that ensures efficient communication between the ER and mitochondria, thereby maintaining intracellular Ca^2+^ homeostasis in the cardiomyocytes. This means that any age-related functional decline of an aging heart, namely changes to mechanical contractility and electrical conductance, are attenuated by the presence of the Cisd2 protein.

### 3.5. Cisd2 Maintains the MAMs during Cardiac Ageing

Normal functioning of MAMs is associated with there being an appropriate width of the cleft between the mitochondrial outer membrane (OMM) and the ER [62]. Certain structural arrangements within the space that separates the ER and mitochondria are able to support an optimal Ca^2+^ transfer via the Ca^2+^ transport channels [46]. Thus, there is an optimal distance of ≈30 nm between ER and mitochondria that allows effective Ca^2+^ transfer from ER, through the Ca^2+^ channel IP_3_R located in ER, to the mitochondria, through the Ca^2+^ channel MCU located in mitochondria. This Ca^2+^ transfer contributes to the generation of physiologically relevant cytosolic Ca^2+^ oscillations [63]. In addition, a previous study has defined the role of this microenvironment as being the key compartment driving cytosolic Ca^2+^ oscillations. The microenvironment between the ER and mitochondria plays a critical role in Ca^2+^ dynamics by modulating the activity of IP3Rs and allowing the shuttling of Ca^2+^ between the ER and mitochondria [64]. A disruption of ER-mitochondria integrity and communication and a consequential dysregulation of Ca^2+^ homeostasis have been linked with pathogenesis, aberrant metabolism [38,42], neurodegenerative disease [38], a decreased lifespan [65], and ageing [15,41].

If the cleft between OMM and ER is too narrow, this causes various abnormalities. For example, under ER stress, MAMs become closer to the mitochondria, with a 25% decrease in distance and a 60% increase in contact length between the two organelles [39]. This results in an enhanced transport of Ca^2+^ from the ER to the adjacent mitochondrial network, which stimulates oxidative metabolism and induces the apoptotic program [16,34]. Additionally, during long-term cultured neuron, which is a neuronal senescence model, an increased Ca^2+^ transport from the ER to mitochondria has been associated with an upregulation of the MCU [66]. Thus, it seems that increased Ca^2+^ transfer to mitochondria and an accumulation of Ca^2+^ within the mitochondria seems to serve as one of the mechanisms underlying the loss of mitochondrial membrane potential and the induction of cell senescence [67].

If the cleft between the OMM and ER is too wide, this also causes problems. A previous study has revealed that abnormally high cytosolic Ca^2+^ develops when the distance is greater than the optimum [63]. Remarkably, our studies have revealed that Cisd2 deficiency or a decreased expression of Cisd2 to less than 50%, namely haploinsufficient, disrupts Ca^2+^ homeostasis and causes elevated cytosolic Ca^2+^ levels. This Cisd2-mediated Ca^2+^ dysregulation is accompanied by abnormal dilation of the ER and a breakdown of the mitochondrial outer membrane [17,18,19] in many different tissues and organs, including brown adipose tissue [68], skeletal muscles [22,53], during neurodegenerative Alzheimer’s disease [23], and during cardiac dysfunction [19].

In naturally aged WT mice at 24-mo old, in which the level of Cisd2 has decreased to less than 50% in the heart, and in prematurely aged Cisd2KO mice at 3-mo old, in which Cisd2 expression is completely eliminated in the heart, the MAM gap distance is obviously increased (Figure 2B); this seems to provide an explanation for the differences in cardiac metabolism and Ca^2+^ signaling that occur with these two mouse models [19]. Intriguingly, in the long-lived Cisd2TG mice at 24-mo old, in which Cisd2 is 2-fold higher in the heart, the compactness and proximity of the ER and mitochondria appears to be well preserved (Figure 2B). This probably ensures that there is correct contact of the mitochondria with the MAM, which then allows normal Ca^2+^ trafficking between these two organelles to be maintained. The cardiac phenotypes of Cisd2KO mice and Cisd2TG mice are summarized in Table 1.

## 4. Metabolic Reprogramming during Cardiac Ageing

### 4.1. Metabolic Reprogramming during Ageing and Its Relationship with Age-Related Cardiac Dysfunction

In the normal myocardium, the heart relies on cardiac mitochondria to generate 90–95% of the ATP it needs via mitochondrial oxidative phosphorylation (OXPHOS) and then uses this ATP to maintain the cardiac functioning [69,70,71]; only 5–10% of ATP needed is generated by anaerobic glycolysis in the cytosol [72]. The ATP generated from OXPHOS is classified as being 40–60% from fatty acid oxidation (FAO), 20–40% from carbohydrate metabolisms via pyruvate and lactate, and 8–10% from ketone body metabolism (of the last, β-hydroxybutyrate and acetoacetate account for 2.6% ± 0.3% and 6.0% ± 1.0%, respectively) [9]. Under different conditions, the energy supply from fatty acids and carbohydrates can be dynamically adjusted, which has been named heart metabolic flexibility [59]. Cardiac mitochondria play an important role not only in biogenesis, but also in the metabolic balance between glycolysis and FAO. In addition, the “creatine kinase-phosphocreatine (PCr) energy shuttle” is pivotal to the energy transfer and storage of such large amount of a high-energy phosphate compound as ATP [73].

The ageing hearts usually shift their energy resources toward a greater reliance on glycolysis and ketone body oxidation and this is accompanied by a decrease in the contribution of mitochondrial OXPHOS. This can aggravate cardiac dysfunction and bring about a progression towards heart failure during ageing [9,15]. During ageing, FAO is decreased and glucose metabolism is enhanced, which leads to lipid accumulation and cell death in the ageing heart, accompanied by fibrosis. Additionally, previous studies have demonstrated that the ATP/PCr ratio is significantly reduced in a failing human myocardium. The efficiency of energy production and of high-energy phosphate metabolism also decline with age [10]. This decline in cardiac metabolic functioning and the associated reprogramming of the heart’s energy metabolic pathways are probably good targets for the development of novel drugs that are able to delay cardiac ageing and treat age-related cardiac diseases.

### 4.2. Understanding the Mechanisms behind the Metabolic Reprogramming of the Ageing Heart

Defects affecting cardiac metabolic functions are mainly caused by (1) impaired FAO and increased lipid accumulation, (2) reduced glucose oxidation and increased pentose phosphate pathway (PPP), (3) inefficient ketone body metabolism, and (4) mitochondrial dysfunction and diminished OXPHOS. Together, these age-dependent dysregulations in energy metabolism and the loss of metabolic flexibility lead to reduced ATP production and result in an inadequate energy supply fueling cardiomyocytes. This then leads to cardiac dysfunction and the development of age-related cardiac diseases in an ageing heart.

#### 4.2.1. Reduced Glucose Oxidation and Increased PPP

**Figure 3:**
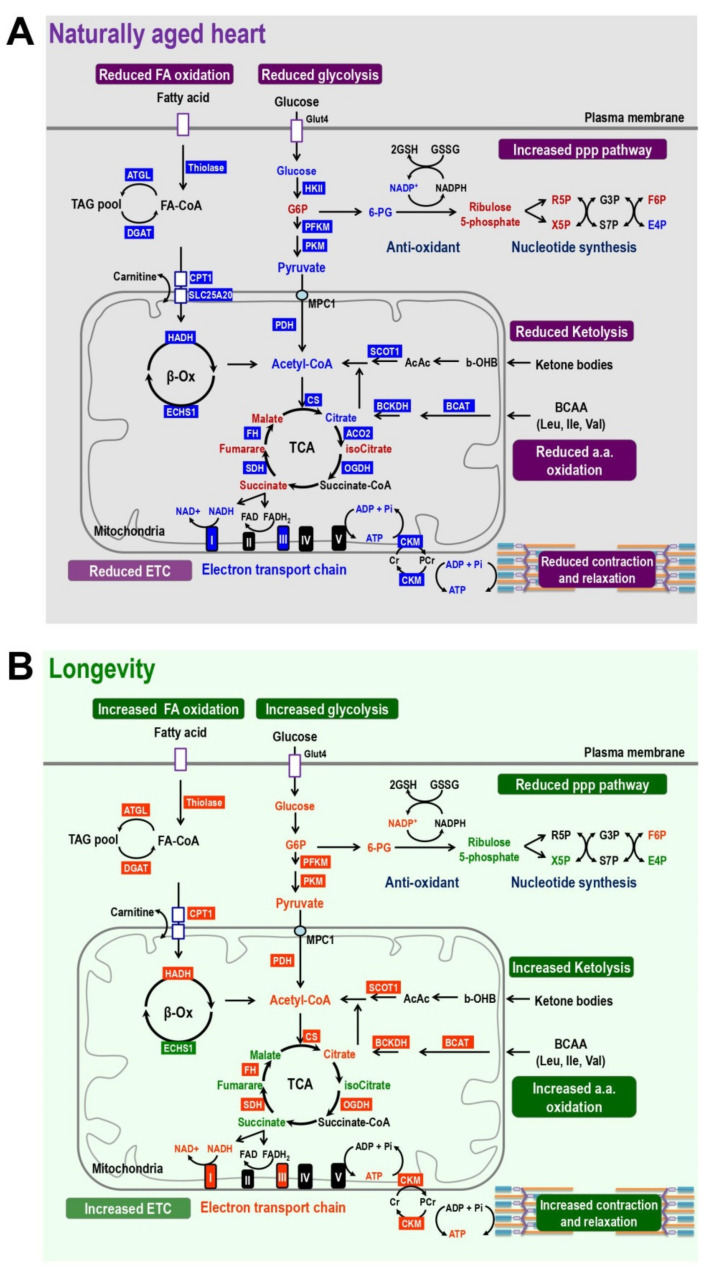
Cisd2 maintains a younger pattern of energy metabolism which is dysregulated in naturally aged heart. (**A**) Energy metabolism is disrupted during natural aging of wild-type (WT) mice. The results are obtained by comparing 24-mo WT vs. 3-mo WT mice. In the aged heart, the TCA cycle and ATP production are impaired due to decrease of fatty acid oxidation, ketolysis, glucose utilization, and amino acid oxidation. In addition, the creatinine shuttling of high-energy phosphate is also diminished in the aged heart. Blue and red colors mark the enzymes and metabolites that are downregulated or upregulated in the pathways, respectively. (**B**) Cisd2 maintains a younger pattern of energy metabolism in the heart of Cisd2TG mice, which is a long-lived mouse model. The results are obtained by comparing 24-mo Cisd2TG vs. 24-mo WT mice. Remarkably, in the Cisd2TG heart, there is an increase in the fatty acid oxidation, ketolysis, glucose utilization, and amino acid oxidation. Consequently, this appears to result in a normal cycling of the TCA and an increased activity of the electron transport chain to produce ATP. In addition, the creatinine shuttling of high-energy phosphate is also restored in the longevity heart. All together, these beneficial effects brought about by Cisd2 provide a sufficient energy supply to fuel the heart of the Cisd2TG mice. The levels of the enzymes and metabolites which have an opposite profile to that observed in the naturally aged heart are marked by green (down) and orange (up). In this study, alterations of metabolic pathways are summarized from omics analyses of metabolomics and transcriptomics of RNA sequencing using cardiac tissues of left ventricles.

In the aging hearts, energy metabolism switches from the utilization of fatty acids toward using glucose as an energy source. The glycolytic rate is increased and this is likely brought about by the increase in activity of ATP-dependent 6-phosphofructokinase (PFK1), the rate-limiting enzyme of glycolosis [74]. However, the whole glycolytic pathway is impaired. As a result, the downstream TCA cycle and the mitochondrial ETC both have diminished activity due to an insufficient supply of electron carriers, namely NADH and FADH_2_, entering the ETC and generating ATP [74,75,76,77,78,79,80,81]. Therefore, although the amount of glucose utilization is increased, the absolute amount of ATP produced by glucose oxidation declines (Figure 3A). Previously Randle and colleagues have proposed a “glucose-fatty acid cycle” to describe fuel selection by various tissues. They demonstrated that the utilization of one nutrient, namely glucose or fatty acid, is able to inhibit the use of the other directly and without hormonal mediation [82]. This Randle cycle of glucose-fatty acid competition is thus a biochemical mechanism for controlling fuel selection. This seems to provide a possible explanation, at least in part, as to why the utilization of glucose is significantly elevated in the ageing heart when fatty acid metabolic pathways are impaired.

In addition to the energy producing pathways, the pentose phosphate pathway seems also to be upregulated in the ageing heart. Previous studies have found that the activity of the key enzyme in this pathway, glucose-6-phosphate dehydrogenase (G6PD), is increased when cardiac hypertrophy is present [83,84]. In the naturally aged heart, our results have revealed that the metabolites G6P and ribulose 5-phosphate are significantly increased compared to young hearts (Figure 3A).

#### 4.2.2. Inefficient Ketone Body Metabolism

Ketone bodies (KB), namely accumulated acetoacetate, beta-hydroxybutyrate, and acetone, are generated in liver via the ketogenesis pathway and are then released into plasma as fuel for many organs including the brain, heart, and muscle [85]. Since the consumption of free fatty acids is decreased in the ageing heart or in a heart with an age-related disease, ketone bodies are considered to be an “energy-efficient fuel” that produces more ATP from a small pool of substrates. Accumulating evidence has indicated that a ketone diet may have a beneficial effect on age-related diseases, and that cyclic ketone bodies probably have the ability to preserve the “young cardiac phenotype” in old mice [85,86,87,88]. Furthermore, in both mouse models and human patients, cardiomyocytes switch to ketone metabolism for oxidative ATP production when there is heart failure and hypertrophy [89,90]. Moreover, treatment with the cardiac protective compound empagliflozin has been shown to increase the level of ketone bodies in the plasma and to improve heart failure [91]. This further supports the cardioprotective role of ketone bodies. Therefore, elevated levels of ketone bodies in plasma and the utilization of ketone bodies by cardiomyocytes seem to enhance ATP production and improve cardiac functions in ageing hearts.

Notably, although ketone body metabolism is able to produce more ATP and support the ageing heart, the expression of a key enzyme, SCOT, has been found to decrease with age (Figure 3A). This indicates that the metabolism of ketone body remains less efficient in the older heart compared to the young heart. Indeed, increasing ketone oxidation in the heart, via enhanced levels of SCOT or other regulatory enzymes involved in ketolysis, has been proposed as a therapeutic target for improving cardiac functioning in a failing heart or when there is age-related cardiac dysfunction [92].

#### 4.2.3. Mitochondrial Dysfunction, Diminished OXPHOS, and Impaired FAO

Fatty acids are the main energy source in a healthy young heart. However, the utilization of fatty acids declines with age [93,94]. In the normal heart, the signaling pathways related to peroxisome proliferator-activated receptors (PPARs) control fatty acid oxidation via targeting of pyruvate dehydrogenase kinase 4 and acetyl-CoA oxidase [95]. Age-related suppression of PPARα results in decreased myocardial fatty acid metabolism and reduced ATP production, and then leads to lipid accumulation [69,96,97,98,99,100]. In addition, the activation of PPARγ signaling results in lipid accumulation and the impairment of FAO-related enzymes in the heart [101,102]. Age-related cardiac dysfunctions are usually accompanied by a decline in FAO rates. However, the uptake of fatty acids remains unchanged or can even be upregulated in some cases, which leads to lipid accumulation in cardiomyocytes [103,104,105]. Indeed, a previous study has showed that the expression of a fatty acid transporter, CD36, is increased in certain elderly populations [106]. An accumulation of lipids can generate toxic lipid species; this in turn has been reported to increase the ROS level and impair mitochondrial functioning. Moreover, such lipotoxicity may trigger inflammation, cell apoptosis, and mitochondrial dysfunction. This can eventually result in a vicious cycle that exacerbates age-related cardiac dysfunction and cardiomyopathy [107,108].

In addition to a loss of metabolism flexibility and enhanced lipid accumulation in the ageing heart, the age-related structural alteration, and functional decline of the component proteins of ETC also contribute to decreased OXPHOS capacity. Complexes I, III, and IV play major roles in the transfer of electrons from NADH to generate the H^+^ gradient for ATP production. During natural ageing of the heart, previous studies have reported that the functioning of complex I and complex III declines [59,109,110,111]. In agreement with this, our transcriptomics analysis of RNA sequencing data has revealed that the mRNA levels of several components of complex I and complex III are significantly decreased in old mice at 24-mo old (Figure 3A).

### 4.3. Cisd2 Delays Cardiac Ageing and Maintains a Younger Metabolism during Old Age

The expression level of Cisd2 decreases during cardiac ageing. Cisd2 deficiency causes cardiac dysfunction and accelerates cardiac ageing. Conversely, a persistently high level of Cisd2 attenuates age-related structural damage and the related functional decline [19]. Intriguingly, in the hearts of Cisd2TG mice at 24-mo old, the metabolic dysregulation in energy production and loss of metabolic flexibility associated with age is significantly improved. Remarkably, the majority of differentially expressed proteins (enzymes or transporters) and their relevant metabolites, including abnormally upregulated and downregulated genes and various metabolites, appear to have their age-associated changes in expression reversed in the heart of Cisd2TG mice, which have a more than 2-fold higher expression level of Cisd2 in their heart compared with age-matched and sex-matched WT mice. We have made the following discoveries based on our analysis of our transcriptomics and metabolomics data. Firstly, the decline in creatine kinase (CKM), which is involved in the PCr energy shuttle, is reversed in Cisd2TG mice; this indicates that the “creatine kinase-phosphocreatine energy shuttle” remains functional and is able to carry out the energy transfer and storage of high-energy phosphate compounds. Secondly, the expression levels of genes involved in the FAO and glycolysis, which are significantly decreased in naturally aged heart, are upregulated in the Cisd2TG mice. Thirdly, the decline in expression level of SCOT, a vital enzyme in ketone metabolism, is reversed in the Cisd2TG mice. Finally, in the Cisd2TG mice, the TCA cycle and mitochondrial ETC (especially complex I and complex III) also show a reversal in their decline compared to aged WT mice (Figure 3B). These findings reveal that a proper regulation of energy metabolism and a dynamic control of metabolic flexibility are retained in the hearts of Cisd2TG mice and thus a higher level of Cisd2 is able to delay cardiac ageing and maintain “a younger cardiac metabolic profile”.

## 5. Conclusions and Perspective

Hundreds of potential antiageing compounds have been reported. Geroprotectors need to fulfill a number of important criteria. Firstly, they should extend lifespan in multiple species and/or different strains of a mammalian animal model. Secondly, they should lessen the human biomarkers of ageing. Thirdly, they need to have minimal side effects and an acceptable toxicity level. Fourthly, they should improve health-related quality of life. Fifthly, they should increase stress resistance. Finally, they should protect against age-related diseases [112,113,114,115]. Many geroprotectors, such as rapamycin, resveratrol, metformin, and various senolytics (e.g., fisetin, dasatininb, and querectin) that are able to remove senescent cells have been reported to improve age-related phenotypes in multiple organs/tissues and/or biological process [115,116,117,118,119,120]. The “National Institute on Ageing Interventions Testing Program” has carried out several trials with the aim of finding potential interventions that can extend the lifespan of genetically heterogeneous mice. The compounds tested can be classed into three groups. The first group consists of acarbose [121], glycine [122], and rapamycin [123]; these three compounds are able to increase lifespan in both males and females. The second group consists of aspirin [124], nordihydroguaiaretic acid [124], protandim [121], and 17α-estradiol [121]; these four compounds are able to increase lifespan in males only. Finally, there is a third group of compounds that were found not to have the ability to extend lifespan and these include metformin, curcumin, and simvastatin [112].

**Figure 4:**
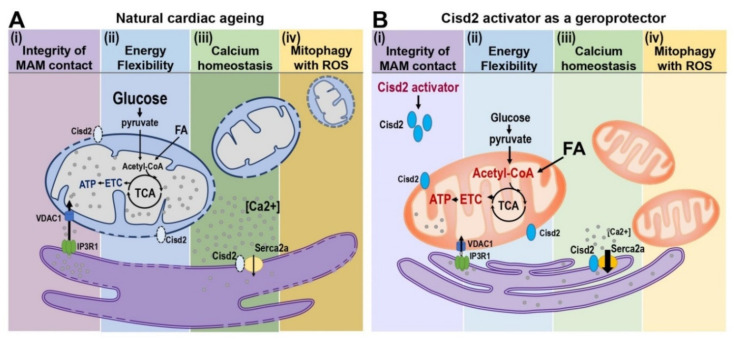
Cisd2 preserves the structure and function of MAM during cardiac ageing. (**A**) Four characters of mitochondria-related dysfunction are identified during cardiac ageing: (i) Disruption of integrity of MAM contact sites. Physical proximity and functional interplay between mitochondria and SR are disturbed during cardiac ageing; this is associated with a widened gap distance between ER and mitochondria leading to an abnormal Ca^2+^ signaling and lipid transport. (ii) Dysregulation of energy flexibility. More than 95% of cardiac ATP is produced from the oxidative phosphorylation of mitochondria via electron transfer chain (ETC), which is fueled with energy primarily from β-oxidation of fatty acids (FA) and to a lesser extent from glycolysis of glucose. Defective fatty acid oxidation of mitochondria jeopardizes the energy flexibility of cardiomyocytes. (iii) Ca^2+^ dyshomeostasis. Elevated cytosolic Ca^2+^ levels resulted from the defective Ca^2+^ recruitment of ER and decreased Ca^2+^ influx capacity of mitochondria as well as impaired cellular Ca^2+^ extrusions are detectable in aged hearts. (iv) Disturbance of mitophagy accompanied with increased ROS. Decrease in the capacity of lysosomal degradation pathway and mitochondrial biogenesis, as well as decrease in mitophagy are observed in aged hearts. Furthermore, these defects usually associated with increased oxidative stress. (**B**) Cisd2 activator as a geroprotector to increase Cisd2 and to preserve cardiac function during ageing. Cisd2 is mainly located in the ER, mitochondria, and MAM. The expression level of Cisd2 decreases during cardiac ageing. A high level of Cisd2 achieved by treatment of Cisd2 activators probably can attenuate these abnormalities during cardiac ageing.

The prolongevity gene Cisd2 is a novel target for geroprotectors and such an approach ought to promote healthy ageing and prevent age-related diseases (Figure 4). Our previous studies and the new evidence provided here highlight Cisd2 as a novel drug target when developing therapies to delay cardiac ageing and ameliorate age-related cardiac diseases.

## Figures and Tables

**Table 1:** Compare the cardiac phenotypes of WT, Cisd2KO and Cisd2TG mice.

Age	Young Age (3 Months Old)		Old Age (24–26 Months Old)	
Genotype	WT	Cisd2KO	WT	Cisd2TG
Genetic modification	-	Conventional Cisd2 KO	-	Carrying 4 copies of Cisd2 gene
Cisd2 protein level	100%	0%	50%	100-130%
Cardiac mechanical function (Ejection Fraction)	~85%	~80% (~70% at 6 months old)	~60%	~75%
Cardiac electrical function	Sinus rhythm	Frequent APCs ^1^ Frequent VPCs ^6^ Atrioventricular block Prolonged Tpeak–Tend interval Prolonged corrected QT interval	Frequent APCs ^1^ Frequent VPCs ^6^ Atrioventricular block Prolonged Tpeak–Tend interval Prolonged corrected QT interval	Rare APCs ^1^ Rare VPCs ^6^
Histopathology	Rare lipofuscin	Increased interstitial fibrosis Increased lipofuscin	Increased interstitial fibrosis Increased lipofuscin	Mild increased interstitial fibrosis Mild increased lipofuscin
Ultrastructure of cardiomyocyte	Normal	Mitochondrial degeneration SR ^5^ dilatation Increased Gap between MAM ^3^ Gap junction extension and fragmentation Myofibril disorganization Fascia adherens breakdown Desmosome degeneration	Mitochondrial degeneration SR ^5^ dilatation Increased Gap between MAM ^3^ Myofibril disorganization Disorganization of intercalated disc Expanded intercellular space	Relatively normal or mild damage
Calcium homeostasis	Normal	Decreased Serca2a activity Decreased [Ca^2+^]_Cyt_^2^ Decreased [Ca^2+^]_SR_^5^ Increased [Ca^2+^]_Mito_^4^	Decreased Serca2a activity	Mild decrease of Serca2a activity
Mito ^4^ function (OCR)	100%	61%	Not determined	Not determined
Oxidative stress (fold increase)	1.0	2.3	2.6	1.2

^1^ APC, Atrial Premature Contractions; ^2^ Cyt, Cytosole; ^3^ MAM, Mitochondria-Associated ER Membrane; ^4^ Mito, Mitochondria; ^5^ SR, Sarcoplasmic Reticulum; ^6^ VPCs, Ventricular Premature Contractions.

## Abbreviations

Glut4	Glucose transporter type 4
HKII	Hexokinase II
G6P	Glucose-6-phosphate
PFKM	ATP-dependent 6-phosphofructokinase
PKM	Pyruvate kinase
MPC1	Mitochondrial pyruvate carrier 1
PDH	Pyruvate dehydrogenase
CS	Citrate synthase
ACO2	Aconitase 2
OGDH	Oxoglutarate Dehydrogenase
SDH	Subunit of succinate dehydrogenase
FH	Fumarate Hydratase
NAD+	Nicotinamide adenine dinucleotide
NADH	Nicotinamide adenine dinucleotide
FAD+	Flavin adenine dinucleotide
FADH2	Reduced flavin adenine dinucleotide
ETC	Electron transfer chain
ATP	Adenosine triphosphate
ADP	Adenosine di-phosphate
6-PG	6-phosphogluconate
R5P	Ribose 5-phosphate
X5P	Xylulose 5-phosphate
G3P	Glyceraldehyde 3-phosphate
S7P	Sedoheptulose 7-phosphate
F3P	Fructose 6-phosphate
E4P	Erythrose 4-phosphate
ATGL	Adipose triglyceride lipase
DGAT	Diacylglycerol O-Acyltransferase
CPT1	Carnitine palmitoyltransferase I
SLC25A20	Solute Carrier Family 25 Member 20
HADH	Hydroxyacyl-coenzyme A dehydrogenase
ECHS	Enoyl-CoA hydratase
SCOT1	Succinyl-CoA-3-oxaloacid CoA transferase
BCAA	Branched-chain amino acids
BCKDH	Branched Chain Keto Acid Dehydrogenase E1
BCAT	Branched-chain amino acid aminotransferase
Leu	Leucine
Ile	Isoleucine
Val	Valine
a.a.	Amino acid
AcAc	Acetylacetonate
b-OHB	Beta-hydroxybutyrate
β-Ox	Beta oxidation
GSH	Glutathione
GSSG	Glutathione disulfide
MAM	Mitochondria-associated membrane
ER	Endoplasmic reticulum
ROS	Reactive oxygen species
TEM	Transmission electron microscopy
[Ca^2+^]i	Intracellular Ca^2+^ concentration
MCU	Mitochondrial Ca^2+^ uniporter
NCX	Na+/Ca^2+^ exchanger
TCA cycle	Tricarboxylic acid cycle
IP3Rs	Inositol 1,4,5-trisphosphate receptors
VDAC	Voltage­dependent anion­selective channel
GRP75	Chaperone 75­kDa glucose­regulated protein
RYR	Ryanodine receptor
Gimap5	GTPase of immune-associated protein 5
PNM	Perinuclear mitochondria
SSM	Subsarcolemmal mitochondria
IFMs	Interfibrillar mitochondria
OMM	Mitochondrial outer membrane
OXPHOS	Oxidative phosphorylation
FAO	Fatty acid oxidation
PCr	Phosphocreatine
PPP	Pentose phosphate pathway
PPARs	Peroxisome proliferator-activated receptors
PFK1	ATP-dependent 6-phosphofructokinase
G6PD	Glucose-6-phosphate dehydrogenase
KB	Ketone bodies
